# Genotype-phenotype correlations in a Scottish CADASIL cohort and comparison with sporadic small vessel disease

**DOI:** 10.1093/esj/23969873251381917

**Published:** 2026-01-01

**Authors:** Sam J Neilson, William Boadu, Amith Sitaram, Rosemarie Davidson, Fiona Moreton, David Alexander Dickie, Jesse Dawson, Keith W Muir

**Affiliations:** School of Cardiovascular & Metabolic Health, University of Glasgow, Queen Elizabeth University Hospital, Glasgow, UK; Department of Neurology, Mater Olbia Hospital, Sassari, Sardinia, Italy; School of Cardiovascular & Metabolic Health, University of Glasgow, Queen Elizabeth University Hospital, Glasgow, UK; Department of Clinical Genetics, Queen Elizabeth University Hospital, Glasgow, Scotland, UK; Department of Clinical Neurosciences, Western General Hospital, Edinburgh, Scotland, UK; School of Cardiovascular & Metabolic Health, University of Glasgow, Queen Elizabeth University Hospital, Glasgow, UK; School of Cardiovascular & Metabolic Health, University of Glasgow, Queen Elizabeth University Hospital, Glasgow, UK; School of Cardiovascular & Metabolic Health, University of Glasgow, Queen Elizabeth University Hospital, Glasgow, UK

**Keywords:** Cadasil, MRI, small vessel disease

## Abstract

**Introduction:**

CADASIL is a monogenic inherited cerebral small vessel disease (SVD) caused by a mutation affecting the NOTCH3 gene. Mutation location appears to influence disease severity. We investigated the hypothesis that mutation location modifies phenotype by comparing a CADASIL population stratified by mutation site risk with a cohort of older people with sporadic SVD.

**Patients and methods:**

We included adults with CADASIL and control group from the XILO-FIST trial. We recorded age at first stroke, white matter hyperintensity (WMH) volume, lacunes, cerebral microbleeds and other clinical biomarkers. We divided the CADASIL cohort into (1) two groups NOTCH3 mutations affecting epidermal growth factor-like repeat (EGFr) domains 1–6 (proximal) and EGFr domains 7–34 (distal); and (2) three groups; low, medium and high-risk based on a proposed three-tiered risk stratification.

**Results:**

The CADASIL cohort included 129 people, 57 (44.2%) male, mean age 47.5 ± 11.7 years. The sporadic SVD cohort included 460 people, 317 (68.9%) male, mean age 65.7 ± 8.7 years. The CADASIL proximal group were imaged at younger age, but fewer had hypertension (14.3% v 38.1%) compared to distal mutations. Lacune count and WMH volume differed between low, medium and high-risk CADASIL mutations, and sporadic SVD. Percentage progression of WMH volume was higher in proximal CADASIL (0.26%), than distal CADASIL (0.14%) which was higher than sporadic SVD (0.05%), *p* < 0.001.

**Discussion and conclusion:**

Proximal CADASIL mutations average more extensive WMH, higher lacune count and experienced first stroke at younger age than those with distal mutations. Both groups showed imaging differences compared to sporadic SVD.

## Graphical Abstract

**Graphical Abstract:**
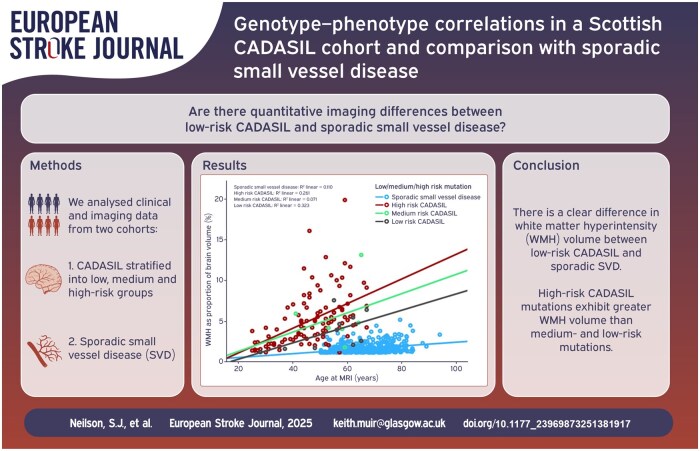

## Introduction

CADASIL (Cerebral autosomal dominant arteriopathy with subcortical infarct and leukoencephalopathy) is a monogenic inherited cerebral small vessel disease (SVD) caused by characteristic mutations of the NOTCH3 gene. Causal mutations modify the number of cysteine residues in the epidermal growth factor-like repeats (EGFr) in the extracellular domain of the NOTCH3 protein, a transmembrane receptor which is expressed in vascular smooth muscle cells^1^ and pericytes and has a role in regulation of vascular function.^2^ CADASIL is associated with stroke and dementia, but both clinical course and MRI findings vary widely among individuals, even within families.^3^ Early studies were unable to establish clear associations between NOTCH3 mutation site and disease severity defined by incidence of stroke, dementia, disability or extent of MRI white matter abnormality.^4^ Coincident occurrence of conventional vascular risk factors such as smoking or hypertension have been associated with earlier manifestation of stroke and vascular dementia,^5^ and higher diastolic blood pressure^6^ and hypertension with the extent and rate of progression of MRI abnormalities.^7^ These factors alone do not completely account for the wide phenotypic variation observed in clinical practice.

Recent population exome analysis identified a much higher prevalence of cysteine-modifying NOTCH 3 mutations^8^ than expected from estimates of CADASIL disease prevalence based on numbers of mutation carriers estimated from pedigrees of clinically affected individuals.^9,10^ This unexpected observation was consistent with a clinical trend for CADASIL being diagnosed more often later in life and with more minor symptoms compared to the classically recognised disease pattern from early CADASIL cohorts.^11,12^ Following the observation that NOTCH3 mutations that modify cysteine residues of EGFr domains 7–34 were highly prevalent in the general population,^8^ but were associated with a lower burden of MRI abnormalities compared to NOTCH3 mutations affecting EGFr domains 1–6, Rutten et al. proposed a dichotomous model of cysteine-affecting NOTCH3 mutations, dividing these into proximal mutations (affecting EGFr domains 1–6) and distal mutations (affecting EGFr 7–34). Proximal mutations were associated with a more severe phenotype consistent with traditional descriptions of CADASIL, while distal mutations were associated with a milder clinical phenotype^13,14^ and less extensive WMH on MRI.^13^ The genotype phenotype correlation has been corroborated in analyses from a UK Biobank sample of over 20,000 people.^15^ UK Biobank genotyping found a prevalence of cysteine-altering NOTCH3 variants of almost 1 in 450. The presence of cysteine-altering NOTCH3 variants was associated with increased risk of stroke and dementia, as well as increased WMH volume.^15^ A cohort of 485 people with CADASIL found proximal mutations associated with earlier onset stroke, younger age of encephalopathy and suggested EGFr domains 10–17 were associated with relatively low stroke risk.^16^ A separate study further divided the EGFr domains seeking to ascertain specific more distal mutations of relatively high risk and proposed a risk stratification system divided into low, medium and high risk mutations based on both clinical manifestations and imaging biomarkers.^17^

We sought to validate genotype-phenotype correlations in a Scottish CADASIL cohort based on clinical and imaging features. We additionally undertook comparisons with a population of presumed sporadic cerebral SVD who had been recruited to a post-stroke randomised controlled trial (XILO-FIST).^18^ This comparison provides novel insights quantifying the degree of imaging differences between more conventional sporadic small vessel disease and those with CADASIL distal mutations.

## Methods

### Study cohort

This is a retrospective cohort study including adults with a CADASIL diagnosis with a pathogenic cysteine-altering NOTCH 3 mutation on genetic testing. All subjects had been reviewed in a neurovascular clinic providing service to the whole of Scotland over a 20-year period from 1999 to 2019. Clinical and brain imaging access was approved by the NHS Safe Haven (GSH/18/NE/007) and Caldicott guardian. Exclusion criteria were contraindications to MRI. We included all cases for whom at least one MRI scan was available for analysis. Scans were undertaken predominantly for routine clinical assessment. Some scans were part of a clinical research study (local research ethics committee reference (12/WS/0295)^19^ for which participants provided written informed consent including for secondary analyses. If participants had more than one MRI performed with an interval of greater than 12 months we included them in a retrospective longitudinal study. The control data set was derived from XILO-FIST, a clinical trial that randomised patients to receive allopurinol or placebo to investigate effects on WMH progression.^18^ XILO-FIST included adults over 50 years of age, with a history of stroke or TIA associated with relevant ischaemic brain lesion on MRI within the previous 4 weeks.^18^ No NOTCH3 genetic testing was performed. Exclusion criteria included presence of dementia, modified Rankin scale score of 5, cognitive impairment compromising capacity and significant renal or hepatic impairment.^18^ This study received approval from the West of Scotland NHS Research Ethics Committee (reference 14/WS/0113). All participants provided written informed consent. This study was performed in accordance with the ethical standards laid down in the 1964 Declaration of Helsinki and its later amendments.

### Clinical data

The following data were collected for all participants with CADASIL: demographics, sex, age at time of MRI, presence of hypertension, diabetes, hypercholesterolaemia, migraine, stroke, antiplatelet prescription, statin prescription, smoking status and alcohol history. Genetic mutation site, age at onset of first stroke and previous encephalopathy were recorded. Clinical data for the control group of sporadic small vessel disease had available age, sex, smoking history, diabetes, hypertension, hypercholesterolaemia.

### Brain imaging

For inclusion in the CADASIL analysis, the minimum requirement for imaging was T2 Fluid attenuation inversion recovery (FLAIR) for white matter hyperintensity measurement and lacune review; -T2 for assessment of enlarged perivascular spaces (EPVS); and T2*- susceptibility-weighted imaging (SWI) or Gradient-echo (GE) imaging for cerebral microbleed analysis. Where multiple T2*weighted sequences were performed preference was given first to SWI then GE. Most scans were performed on clinical 1.5 Tesla scanners, with some on 3 Tesla scanners. Most scans included three-dimensional (3D) T1-weighted and T2-weighted images.

### Image processing

For the CADASIL cohort images were transferred from clinical Picture Archiving Communications System (PACS) to an NHS data Safe Haven to facilitate analysis on a secure platform.

White matter hyperintensities, lacunes, cerebral microbleeds and enlarged perivascular spaces (EPVS) were defined as reported in the STRIVE criteria for imaging standards.^20^ Number of lacunes was recorded. Cerebral microbleeds were counted using the Microbleed Anatomical rating scale (MARS).^21^ EPVS were assessed using the comprehensive instructions on Enlarged perivascular spaces (EPVS): a visual rating scale user guide.^22^ Mango imaging software http://ric.uthscsa.edu/mango/index.html was used for measurements and for visual review of images.^23^ We used the Brain extraction tool (BET) plugin based on the FMRIB Software Library programme to remove non-brain tissue from the initial image of the whole head.^24^ This allowed for measurement of brain volume. We then used the FLAIR sequence areas of hyperintensity in the brainstem and white matter to create a region of interest (ROI) creating thresholds. This was further visually inspected and modified to ensure the inclusion only of subcortical white matter hyperintensities. The volume of these WMH was calculated and an associated Fazekas score was recorded. WMH volume measurements were performed on two separate occasions by one reader (SN) blinded to genetic abnormality, more than 1 month apart. These methods were used for all baseline scans in CADASIL and sporadic small vessel disease cohorts. For the longitudinal study the imaging features of WMH, lacunes and cerebral microbleeds (CMBs) were measured on both scans and interval progression time was calculated. To establish annual rate of progression for each imaging marker the difference in imaging marker was divided by interval time to calculate annual progression. These values were compared to results from a longitudinal analysis of the sporadic small vessel disease cohort analysed as detailed elsewhere.^18^ This analysis used WMH volumetric assessment using atlas-based segmentation creating a probability map in which voxels with ⩾1.5 times standardised *z* score were defined as WMH.^18^

### Statistical analysis

Statistical analysis was performed using IBM SPSS version 29 (IBM, Armonk, New York, USA).

Intra-class correlation coefficient (ICC) was used for inter- and intra-rater reliability. Normality of data was tested using the Shapiro-Wilks test. Differences in baseline clinical characteristics were compared between CADASIL divided both into dichotomous proximal (EGFr 1–6) and distal (EGFr 7–34) subgroups and also into the suggested three-tier risk stratification categories (low, medium and high-risk).^17^ These were compared to the sporadic small vessel disease comparison cohort. The differences between these characteristics including age and vascular risk factors were calculated using independent sample *t*-test for normally distributed continuous variables and Chi-squared or Fisher’s exact test for categorical variables. For clinical characteristics we reported valid percentages excluding unkown cases. A Mann-Whitney *U* test was used to compare difference between ordinal variables and where distribution was not normal. A one-way ANCOVA was used to assess group differences with Bonferroni adjustment. Kruskal-Wallis tests were used for non-normally distributed ordinal data between three or more groups with post hoc comparisons using Dunn’s pairwise test^25^ and Bonferroni correction.

Cox regression analysis was used to compare age at first stroke in the CADASIL cohort between proximal and distal mutations, adjusting for hypertension, diabetes and current or past smoking history. The alpha level for statistical significance was *p* < 0.05.

## Results

### Baseline clinical characteristics

A total of 129 patients with CADASIL were included, of whom 108 (83.7%) had proximal mutations. Of these, 74 individuals (57.4% of whole group) had pathogenic variants affecting the EGFr domains 3 and 4 Figure A (Appendix). The 21 (16.3%) individuals with distal mutations were distributed as shown in Figure A. The comparison sporadic small vessel disease cohort included 460 patients with conventional stroke risk factors.

The summary clinical and imaging differences between CADASIL proximal mutations, distal mutations and sporadic small vessel disease are shown in Table 1. Both CADASIL groups were imaged at younger age – proximal (46.8 ± 11.5 years) and distal (53.5 ± 11.4 years) – compared to sporadic small vessel disease (65.7 ± 8.7 years). Hypertension (47.8%) and diabetes (21.5%) were more prevalent in the sporadic small vessel disease cohort.

**Table 1.** Summary characteristics, clinical and imaging features of Scottish CADASIL population and cohort with sporadic small vessel disease.

Variable	EGFR 1–6	EGFR 7–34	Sporadic small vessel disease	*p*-Value
n	108	21	460	
	Clinical features			
Age at MRI (years)	46.8 ± 11.5	53.5 ± 11.4	65.7 ± 8.7	<0.001
Male/female (% male)	46/62 (42.6)	11/10 (52.4)	317/143 (68.9)	<0.001
Smoking history, *n* (%)	36 (39.1) 16 unknown	10 (59.6) 3 unknown	245 (53.3)	0.044
Hypertension history, *n* (%)	15 (14.3) 3 unknown	8 (38.1)	220 (47.8)	<0.001
Total cholesterol (mmol/l) mean ± SD	4.5 ± 1.0	4.5 ± 1.2	3.9 ± 1.0	<0.001
Diabetes, *n* (%)	7 (6.9) 6 unknown	2 (10.5) 2 unknown	99 (21.5%)	<0.001
Depression, *n* (%)	54 (50.5) 1 unknown	6 (35.3) 4 unknown		0.245
Migraine, *n* (%)	66 (65.3) 7 unknown	11 (73.3) 6 unknown		0.541
Stroke, *n* (%)	47 (46.5) 7 unknown	9 (50%) 3 unknown		0.786
Age at first stroke (years, median [IQR])	49 [43–55]	54 [39.5–63]		0.312
Previous encephalopathy (%)	8 (7.4)	1 (4.8)	-	0.588
Age at first encephalopathy	47 [42–52]	49	-	0.750
	Imaging features			
Brain volume (ml) mean ± SD	1489.1 (188.6)	1517.8 (184.4)	1477.4 (157.1)	0.772
WMH (% of brain volume) median [IQR]	3.5 [1.6–5.5]	2.9 [1.0–4.5]	0.2 [0.1–0.7]	<0.001
Fazekas periventricular median [IQR]	3 [2–3]	2 [2–3]	1 [1–2]	<0.001
Fazekas deep white matter median [IQR]	3 [2–3]	3 [1.5–3]	1 [1–2]	<0.001
Fazekas total median [IQR]	5.5 [4–6]	5 [3.5–6]	3 [2–4]	<0.001
Lacune count median IQR]	3 [0-8.5]	1 [0 -5.5]	1 [0–2]	<0.001
CMBs count median [IQR]	0 [0–2]	0 [0–1]	0 [0–1]	0.235
EPVS score median [IQR]	3 [3–4]	4 [3–5]	4 [3–5]	0.250

### Reliability of measurements of small vessel disease imaging markers

For WMH volume measurements performed by index rater (SN) intra-rater reliability was ICC = 0.920 (confidence interval (CI) 0.814–0.967), *p* < 0.001. These values were compared to another rater values for WMH volumes using the larger validation cohort analysis performed previously,^18^ intraclass correlation coefficient between these values was 0.755 (CI 0.711–0.793), *p* < 0.001. Measurement of CMBs was compared between two raters (SN and AS) for a subset of 20 control cases establishing an inter-rater agreement of 0.909 (95% CI 0.779–0.962), *p* < 0.001.

### Comparison of CADASIL proximal and distal mutations with sporadic small vessel disease

**Figure 1.**
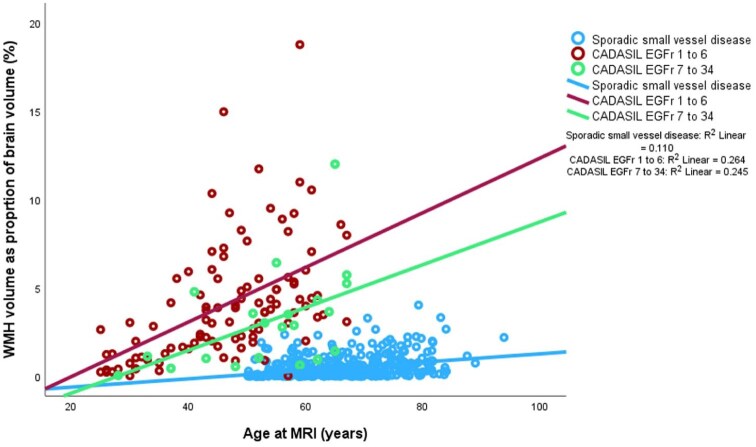
WMH volume as proportion of brain volume (%) versus age in CADASIL proximal and distal mutations and sporadic small vessel disease with linear fit lines.

Fazekas score, WMH volume and lacune number were highest in the CADASIL proximal mutation group followed by CADASIL distal mutations and in turn sporadic small vessel disease (Figure 1), despite the higher age of the sporadic small vessel disease cohort (Table 1). There was no significant difference between cerebral microbleeds and EPVS between the three groups. Lacune count was higher in the proximal CADASIL group than distal CADASIL and sporadic small vessel disease. Using WMH as percentage of brain volume as dependent variable and age as a covariate, we found a statistically significant between-group difference (*p* < 0.001; Table 1). The age-adjusted mean values were 5.0 (95% CI 4.7–5.4) for proximal CADASIL mutations, 3.6 (95% CI 3.0–4.3) for distal CADASIL mutations and 0.2 (95% CI 0.1–0.4) for sporadic small vessel disease.

### Imaging analysis of high, medium and low risk CADASIL with cohort of sporadic small vessel disease

**Figure 2.**
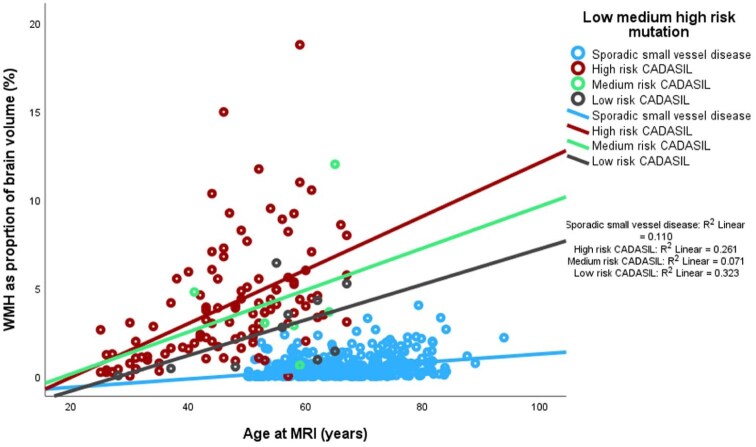
WMH as proportion of brain volume (%) versus age in high, medium and low risk CADASIL and sporadic small vessel disease with linear fit lines.

We perf compared high, medium and low risk CADASIL cases based on the proposed classification of Hack et al.^17^ with the cohort with sporadic small vessel disease (Table 2). Figure 2 shows WMH as proportion of brain volume (%) against age in the low, medium and high-risk CADASIL groups and sporadic small vessel disease.

**Table 2.** Imaging features of CADASIL cohort divided into three-tier risk strata (high, medium and low) and sporadic small vessel disease. All values are median [IQR].

MRI feature	High (113)	Medium (6)	Low (10)	Sporadic small vessel (460)	*p*-Value
WMH (% of brain volume)	3.5 [1.6–5.5]*	3.3 [2.3–6.6]*	2.1 [0.5–4.5]*	0.2 [0.1–0.7]*	<0.001
Lacune count	3 [0–8]*	0.5 [0–2]	0.5 [0–9]	1 [0–2]*	<0.001
CMBs count	0 [0–2]	0 [0–1]	0 [0–3.5]	0 [0–1]	0.419
EPVS score	3.5 [3–4]	5 [4.5–5.5]	4 [3–5.25]	4 [3–5]	0.124

From post-hoc comparisons for each biomarker * indicates groups with statistically significant difference.

### Stroke free survival analysis comparison EGFr domains 1–6 and EGFr domains 7–34

**Figure 3.**
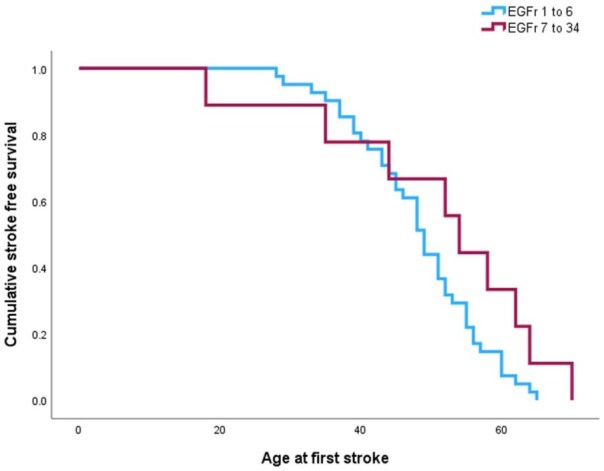
Cox regression event curve for age at first stroke in CADASIL proximal and distal mutations adjusted for hypertension, diabetes and smoking history.

Median age at first stroke was 49 years [IQR 43–55] for proximal mutations and 54 years [39.5–63] for distal mutations, which was non-significant *p* = 0.312 (Table 1). Only nine cases of distal CADASIL had history of stroke. Cox proportional hazards regression analysis for age at first stroke comparing mutation site (proximal or distal) and including hypertension, diabetes and smoking as covariates is shown in Figure 3. The hazard curve suggests younger age of first stroke for people with CADASIL proximal mutations, but this was not statistically significant *p* = 0.128.

Differences between proximal and distal mutations are shown in appendix B and included a significantly higher proportion of distal mutations with hypertension (40%) and older age at MRI. There was a non-significantly higher numerical prevalence of smoking (Table B (Appendix)). Differences in annual rate of progression of MRI markers in the CADASIL cohort divided into proximal and distal mutations and comparison with sporadic small vessel disease are detailed in Table 3.

**Table 3.** Longitudinal rate of change of MRI imaging markers in CADASIL and sporadic small vessel disease. All results median (IQR).

Parameter	EGFR 1–6	EGFR 7–34	Sporadic small vessel disease	*p*-Value
n	28	10	372	
Annual rate of change WMH (% of brain volume)	0.26 (0.09–0.82)*,**	0.14 (0.06–0.65)*	0.05 (−0.01 to 0.18)**	<0.001
Annual rate of incident lacunes	0.15 (0–1.0)**	0 (0–0.5)	0 (0–0)**	<0.001
Annual rate of incident cerebral microbleeds	0 (0–0.7)**	0 (0–1.0)	0 (0–0)**	<0.001

^*^represents significant difference between subgroup EGFR 1-6 and EGFR 7-34.

^**^represents significant difference between subgroup EGFR 1-6 and sporadic small vessel disease.

The median rate of progression of WMH as percentage of brain volume was significantly higher for both proximal (0.26% annually) and distal (0.14% annual increase) CADASIL mutations compared to sporadic small vessel disease (0.05% per annum; *p* < 0.001, Table 3).

Post hoc pairwise comparisons using Dunn’s^25^ procedure with a Bonferroni correction was used to assess significant between group differences. * and ** represent significant group differences within the table. The very small annual rate of change of CMBs result in statistically significant differences although median values were 0.

Extrapolating the annual change in lacune number resulted in an estimated increase in lacunes of mean 4.3 every 10 years for proximal mutations and 1.9 lacunes every 10 years for distal mutations assuming consistent progression. Similarly an estimated increase in cerebral microbleeds of mean 5.8 every 10 years for proximal mutations and 1.9 every 10 years in distal mutations was estimated.

## Discussion

We sought to validate the reported association between location of pathogenic NOTCH3 mutation and disease severity and to quantify differences between the CADASIL population with distal mutations and sporadic small vessel disease. Our findings corroborate the observation that proximal CADASIL mutations are associated with more extensive WMH volume and higher lacune count, compared with distal mutations affecting EGFR 7–34. When dividing the CADASIL cohort into high, medium and low risk mutations according to the proposed stratification schema of Hack et al.^17^ and comparing with a cohort including sporadic small vessel disease, we found a graded increase in extent of WMH from the smallest volume in sporadic small vessel disease through the risk categories to largest volume among those with high risk NOTCH3 mutations. There was a clear difference between distal CADASIL and sporadic small vessel disease. This suggests there may be benefit in the early identification of this CADASIL cohort to optimise cardiovascular risk factor control with the intention of delaying disease progression. In our longitudinal analysis, rates of WMH progression over time were greater for those with proximal compared to distal CADASIL mutations, with both exceeding progression in sporadic SVD.

The observation of a high frequency of 1 in 300 individuals carrying a potentially pathogenic NOTCH3 mutation in a public exome screening dataset from the Netherlands^8^ provided evidence of a genotype-phenotype correlation. The majority of NOTCH3 mutations coding for EGFR regions 7–34 were associated with lower prevalence of MRI abnormalities and clinical history of stroke or dementia compared with NOTCH3 mutations typically associated with a conventional CADASIL phenotype. A high population prevalence of cysteine -altering NOTCH3 variants was reported in an analysis of UK biobank data of over 200,000 individuals, with prevalence of around 1 in 450.^15^ Previous, much lower, estimates for prevalence of clinical CADASIL cases^9,26^ are consistent with asymptomatic or paucisymptomatic cases being responsible for the majority of potentially pathogenic NOTCH3 mutations. Additional investigation of EGFr location as a risk for disease severity has resulted in the division of CADASIL populations into proximal EGFr 1–6 and distal EGFr 7–34 mutations, with proximal mutations showing a more aggressive disease trajectory.^13^ Our study shows that WMH volumes increase with age in both proximal and distal mutations, but that WMH burden is higher with proximal mutations. The rate of increase in both is higher than that seen in the cohort of patients with recent stroke or TIA participating in the XILO-FIST trial. Our study results suggest more rapid increase of WMH volume in proximal mutations compared to distal CADASIL mutations and in turn sporadic small vessel disease. The extent of WMH burden in the general population is significantly associated with the development of stroke, dementia and death.^27^ The influence of WMH on disease progression in CADASIL is less clearly established, with Jouvent et al.^28^ and Liem et al.^29^ finding no significant association over 3 and 7 years’ follow-up, whereas Ling et al.^30^ noted progression in WMH volume over time and an association between this and decrease in modified Rankin score. Our study population also shows that those with proximal mutations were first imaged at a younger age than distal mutations. Further, the higher proportion with a diagnosis of hypertension in the distal mutation subgroup is consistent with both an interaction of NOTCH3 mutations with conventional vascular risk factors, as suggested previously,^13,31,32^ and with the possible requirement for additional risk factors to result in full clinical penetrance of distal mutations. Cardiovascular risk profile has been associated with greater risk of severe clinical manifestations in CADASIL.^5,33^

A three-tier system of low, medium and high risk CADASIL mutations has been proposed in recognition of higher-risk phenotypes associated with EGFr domains 8, 11 and 26.^17^ Our analysis, although limited by small numbers, suggests that the WMH burden and lacune count follow similar patterns (Figure 2).^17^ A different European cohort recruited through the UK Familial Cerebral Small Vessel Disease Study consisting of 485 patients had genetic mutation reviewed and demonstrated EGFr domain 10–17 were associated with a significantly lower risk of stroke.^16^ Our study population is similar to other European cohorts, with a heavy predominance with abnormality in EGFr domains 3 and 4 (Figure A in Appendix).^17^ This contrasts to studies in Asian populations where abnormalities in EGFr domain 11 appear to be most prevalent.^34,35^

The longitudinal component of this study showed a lower median rate of progression of WMH as a percentage of brain volume compared to previous CADASIL prospective studies (0.19% (0.06–0.76)) annually, compared with 0.28% averaged over 3 years^30^ and 0.29% annually over 2 years.^36^ Our observed progression rate in the CADASIL cohort is higher than our sporadic SVD cohort and that reported for sporadic SVD elsewhere. The increase in WMH volume as a percentage of intracranial cavity volume from a meta-analysis is 0.25% over a mean interval 3.5 years between MRI.^37^ We found higher median WMH rate of progression among proximal mutations compared to distal (0.26% annually compared to 0.14%) although small numbers in distal group (eight cases) limit conclusions. Estimated annual lacune incidence was numerically higher in proximal mutations compared to distal (annual rate median 0.15 vs 0).

Limitations of our study include small numbers of distal or low risk NOTCH3 mutations, consistent with the clinical CADASIL populations. Imaging data were gathered predominantly from routine clinical imaging, undertaken for diagnosis or in response to clinical events. MRI scanners of different field strengths were used at different geographical locations, and without any standardised protocol. Individual scanners operating at lower field strengths may have lesser ability to detect WMH, lacunes, CMBs and EPVS. Despite these factors, the demonstration of similar WMH progression rates to previous studies confirms the feasibility of including MRI analysis outside research protocols. Age at MRI and interval between MRI may correlate with clinical disease severity although we have no outcome measure to confirm this. The comparison between CADASIL proximal and distal mutations and sporadic small vessel disease has potential limitations due to the differences in baseline characteristics. As well, as being older, all participants in the XILO-FIST trial had recent stroke or TIA events and would be on secondary prevention potentially resulting in lower cholesterol levels. However, these cohort differences remain representative of the core populations associated with these differing clinical disease entities. We were able to demonstrate differences in imaging features of all three groups both through the more extensive WMH volume and number of lacunes, but also through the suggestion of more rapid progression of both imaging features longitudinally. This finding, although intuitively likely because of the often very extensive WMH seen in the CADASIL population has not been reproduced widely. The differences in imaging features in this study suggest that the included patients with distal NOTCH3 CADASIL mutations are displaying evidence of a differing clinical syndrome than sporadic small vessel disease. The control population was derived from the XILO-FIST trial, which sought progression of WMH burden as its primary endpoint, but recruitment was not restricted to patients with cerebral SVD and included any stroke or imaging-confirmed TIA in the preceding 4 weeks. The XILO-FIST trial population exhibited a high prevalence of imaging-based cSVD findings that are commonly present across all stroke mechanisms.

Further research is needed to establish the boundaries between people with CADASIL and a distal, low risk mutation or an unaffected individual with an incidental NOTCH3 abnormality without symptoms. Since the XILO-FIST trial population diagnosed SVD based on imaging findings and did not undertake any genetic testing, we cannot exclude the possibility of this cohort including individuals with low or medium risk NOTCH3 mutations.

In conclusion, our findings suggest different clinical and imaging severity in proximal pathogenic NOTCH3 mutations compared to distal mutations in CADASIL and sporadic small vessel disease with a more rapid progression of imaging features. The higher prevalence of hypertension in distal NOTCH3 mutations suggests a potentially greater role for conventional vascular risk factors in the clinical manifestation of a CADASIL phenotype. Both proximal and distal NOTCH3 mutation groups exhibited significantly earlier clinical presentation and more extensive imaging findings than a control group of sporadic SVD. Further research is required to understand the reasons behind the discrepancy between the rarity of clinical manifestations of CADASIL with distal NOTCH3 mutations and the relatively high frequency with which these mutations occur in more general populations.

## Figures and Tables

**Figure A.**
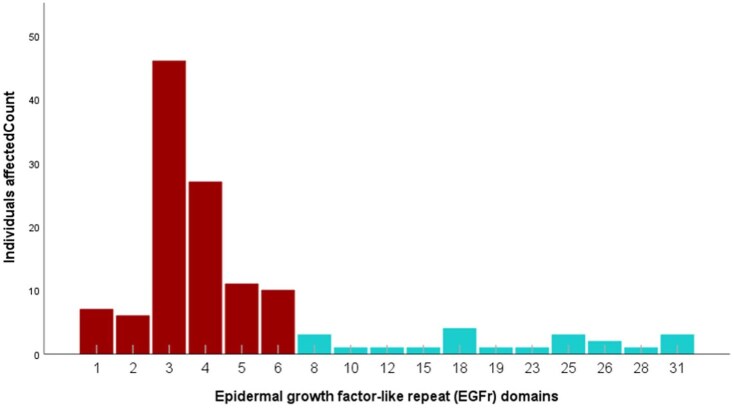
Distribution of CADASIL cases across different EGFr domains proximal mutations (red) and distal (green).

## Appendix

**Table B.** Differences in clinical features in CADASIL cohort in longitudinal retrospective comparison.

Clinical feature	EGFr 1–6	EGFr 7–34	*p*-Value
*n*	28	10	
Age at MRI (mean ± SD)	44.8 ± 11.7	54.3 ± 10.7	<0.001
Smoker, yes/no (%)	7/28 (25)	5/10 (50)	0.144
Hypertension, yes/no (%)	4/28 (14.3)	4/10 (40)	0.010
Diabetes, yes/no (%)	3/28 (10.7)	1/10 (10)	0.721
Migraine, yes/no (%)	16/28 (57.1)	6/10 (60)	0.589
Stroke, yes/no (%)	12/28 (42.9)	4/10 (40)	0.583

## Data availability

Data from this study may be available from the corresponding author upon reasonable request.
